# Seven-Year Comparative Outcomes of One Anastomosis Gastric Bypass and Roux-en-Y Gastric Bypass for Weight Recurrence Regain Post Sleeve Gastrectomy

**DOI:** 10.1007/s11695-026-08824-w

**Published:** 2026-07-23

**Authors:** Abdelwahed Yahmadi, Asaad Salama, Hamzah El Baba, Jawher Baazaoui, Moataz Bashah, Mohammed AlKuwari

**Affiliations:** National Bariatric Center (NBC), Hamad Medical Corporation, Doha, Qatar

**Keywords:** Bariatric surgery, Sleeve gastrectomy, Roux-en-Y gastric bypass, One-anastomosis gastric bypass, Conversion surgery

## Abstract

**Background:**

Sleeve gastrectomy (SG) is currently the most performed bariatric procedure worldwide; however, up to half of patients may require conversion surgery due to insufficient weight loss, weight recurrence, or complications. Roux-en-Y gastric bypass (RYGB) and one-anastomosis gastric bypass (OAGB) are the two most common conversion options, yet evidence regarding long-term comparative outcomes remains limited.

**Objective:**

This study compared the long-term outcomes of RYGB and OAGB after SG, focusing on weight loss, resolution of comorbidities, complication rates, and nutritional deficiencies.

**Methods:**

We conducted a retrospective analytic study of all patients who underwent conversion RYGB or OAGB at our institution between January 2014 and December 2016. Data were extracted from electronic medical records, including demographics, comorbidities, perioperative details, and anthropometric and biochemical measures at baseline, 1, 5, and 7 years. Statistical analysis was performed using SPSS version 26.

**Results:**

109 patients were included (47 OAGB, 62 RYGB). Operative time was significantly shorter for OAGB. Both procedures achieved durable weight loss; however, OAGB yielded superior long-term outcomes, with greater %TWL and BMI reduction at one and seven years (*p* < 0.05). RYGB was significantly more effective for GERD resolution (*p* = 0.002). Rates of diabetes, hypertension, and dyslipidaemia remission were comparable. Complication rates were low and similar, though nutritional deficiencies, particularly iron deficiency, were more common in OAGB.

**Conclusion:**

OAGB provided superior long-term weight loss, while RYGB offered better GERD resolution. Both procedures were safe and effective, underscoring the need for individualised procedure selection.

**Key Points:**

Both OAGB and RYGB demonstrated sustainable weight loss at 7-year follow-up after conversion from SG

OAGB resulted in greater long-term weight loss, whereas RYGB provided better control of GERD symptoms.

No significant differences were observed in terms of comorbidities control.

## Introduction

Obesity has turned into a major public health issue globally due to its rising prevalence and associated comorbidities [1–5]. Lifestyle changes, including behaviour modification and pharmacological interventions, usually have limited, short-term outcomes. This demonstrates the importance of bariatric surgery as the most effective intervention for achieving sustained weight loss and better life outcomes in obesity [6].

Among the several types of bariatric surgery, laparoscopic sleeve gastrectomy (LSG) has recently become the most commonly performed procedure [7, 8]. Its popularity could be attributed to its relative technical ease, shorter operative time, and positive short-term weight reduction and comorbidity remission rates, similar to bypass procedures. In the 2023 International Federation for the Surgery of Obesity and Metabolic Disorders (IFSO) global registry report, LSG accounted for 60.4% of all bariatric surgeries worldwide [9, 10].

However, despite these positive results, long-term follow-up studies have demonstrated that LSG has significant limitations. Some studies have observed that 30–50% of patients may eventually need to undergo conversion surgery because of insufficient weight loss, weight regain, or complications related to the sleeve itself, including but not limited to, severe gastroesophageal reflux disease (GERD), gastric stenosis, dilation, etc.[11–13].

Consequently, conversion surgery following LSG has gained prominence, with Roux-en-Y gastric bypass (RYGB) and one-anastomosis gastric bypass (OAGB) being the most commonly performed procedures in this setting. RYGB is a very common conversion bariatric procedure after SG, with long-lasting weight loss and resolution of comorbidities. However, its technical nature, though, and threats of internal hernia, marginal ulcers, and micronutrient deficiencies should be considered when selecting it as a conversion procedure. OAGB, an evolved modification of the previous “mini-gastric bypass”, is a more recent variation of gastric bypass surgery. Its design as a single gastrojejunal anastomosis, which is technically easier and faster to do. Moreover, establishing a longer biliopancreatic limb provides more potent malabsorptive action, which may translate into more sustained weight loss compared to RYGB [14–16].

Although the literature contains many studies comparing RYGB and OAGB after the unsatisfactory results of LSG, most of them are short or mid-term studies, usually happening within three to five years post-surgery. Long-term comparative data are scarce, even though it is crucial to analyse the durability of the efficiency and safety of conversional procedures. Since obesity is a chronic, relapsing condition, long-term comparative results are valuable for conceptualising the durability of weight loss, remission of comorbidities, and patterns of complications.

Therefore, this study compares the long-term (5- and 7-year) safety of RYGB and OAGB as conversion surgeries following LSG with respect to weight-loss effectiveness and correction of obesity-related comorbidities. Moreover, the secondary goal is to compare the long-term safety charts of the two procedures, with particular emphasis on complication rates and nutritional deficits. This study aims to provide clinically relevant evidence to guide surgeons and patients in selecting the optimal conversion strategy to maximise long-term outcomes, with a focus on both efficacy and safety.

## Methods

This study is a retrospective cohort study conducted at a tertiary bariatric center. Patients were recognised using the hospital’s bariatric surgery database. Their clinical information was accessed in electronic medical records.

No a priori sample size calculation was performed. The sample size was determined by the number of eligible patients meeting the predefined inclusion and exclusion criteria within the study period.

The study included patients who underwent RYGB or OAGB as a conversional procedure for weight recurrence regain, or suboptimal weight loss post-sleeve gastrectomy in the period between January 2014 and December 2016. The time frame was chosen to ensure sufficient follow-up, thereby permitting an assessment of outcomes at seven years post-surgery. Patients who underwent conversion surgery to address sleeve gastrectomy complications were excluded.

Suboptimal outcomes after sleeve gastrectomy were defined according to accepted international standards as either insufficient weight loss—defined as a percentage of total weight loss (%TWL) of less than 20% and/or a percentage of excess weight loss (%EWL) of less than 50% at 18–24 months postoperatively—or significant weight regain following an initial satisfactory weight loss response [6, 7].

This study utilized a predefined analytical cohort comprising patients who underwent conversion from SG to either OAGB or RYGB for weight recurrence regain or suboptimal weight loss after SG during the predefined period; therefore, institutional totals for other revision categories were not included. As this is a retrospective cohort study, the conversional procedure was not randomized but reflected our clinical practice. In general, patients with GERD symptoms were offered RYGB; however, other patients with weight recurrence regain without GERD symptoms had their conversional surgery selected based on the surgeon's advice or the patient's preference, through shared decision-making. The 7-year endpoint was selected because it represented the maximum available follow-up duration for the study cohort for the defined surgical period (2014–2016), enabling assessment of long-term durability beyond the commonly reported 5-year timeframe.

Data collection was conducted systematically and encompassed a broad range of variables. Demographic information included age, sex, and measurements prior to conversion surgery. Clinical variables comprised obesity-related comorbidities like type 2 diabetes mellitus, hypertension, dyslipidaemia, obstructive sleep apnoea, asthma, and gastroesophageal reflux disease. Recorded operative details consisted of the nature of conversion surgery (RYGB or OAGB), time of operation, intraoperative observations, and immediate intraoperative complications. For each patient, we also recorded the length of hospital stay.

Anthropometric measurements were taken at specific intervals: prior to the conversion and subsequently at 1, 5, and 7 years after the conversion procedure. These were body weight, BMI, percentage of excess weight loss, and percentage of total weight loss. The levels of haemoglobin, ferritin, vitamin B12, folates, calcium, and vitamin D were also measured at the same follow-up points to assess nutritional deficiencies. The outcome of comorbidity was assessed as complete remission, partial improvement, or no change/worsening, according to commonly accepted definitions reported in the literature [3, 6, 13, 15]. The complications were divided into early (within 30 days of surgery) and late (after 30 days), and further subdivided into the means of surgical complications (including leak, bleeding, internal hernia, bowel obstruction, stricture, and marginal ulcer) and nutritional complications (including iron deficiency anaemia, vitamin deficiencies, and severe protein-calorie malnutrition).

To reduce information loss, follow-up data were obtained through outpatient clinic visits, laboratory test results, and telephone interviews with patients (where needed). Two investigators reviewed all the data separately to ensure uniformity, and disagreements were settled through consensus.

The Statistical Package of the Social Sciences (SPSS), version 26.0 (IBM Corp., Armonk, NY, USA), was used for statistical analysis. Continuous variables were reported as mean and standard deviation, whereas categorical variables were reported as frequencies and percentages. The t-test of students or the Mann–Whitney U-test was applied to the RYGB vs. OAGB groups to compare the continuous variables according to the normality of data. Categorical variables were tested using the chi-square or Fisher’s exact test. The p-value < 0.05 was deemed significant. Kaplan–Meier survival analysis was applied to evaluate the likelihood of being free of conversion-related complications over time. Cox proportional hazards regression was used to assess predictors of long-term outcomes.

The Institutional Review Board granted ethical approval for this retrospective study, and the study was conducted in accordance with the principles of the Declaration of Helsinki. Since this was a retrospective review of existing records, individual informed consent was no longer required.

### Surgical Technique

All procedures were performed as conversional laparoscopic procedures after SG, and none were converted to open procedures.

### RYGB Procedure

The Roux-en-Y gastric bypass was performed using a standardized technique by creating a 30–50 ml pouch by transecting the sleeved stomach 4 cm below the gastroesophageal junction. The pouch was trimmed in selected cases of dilated or redundant gastric pouch, based on intraoperative assessment. The alimentary limb measured 100 cm, and the biliopancreatic limb 125 cm. Both the Peterson and mesenteric defects were closed systematically. Drains were not utilized.

### OAGB Procedure

A long gastric pouch was created by dividing the sleeved stomach at the level of the incisura angularis. The duodenojejunal junction was then identified, and a jejunal loop measuring approximately 150–200 cm was brought up in an antecolic fashion to establish a side-to-side gastrojejunal anastomosis. The anastomosis was performed either as a totally hand-sewn or a stapled-and-hand-sewn technique, according to the surgeon's individual preference.

## Results

### Baseline Characteristics

The analysis involved 109 patients who had conversional bariatric surgery following sleeve gastrectomy. Forty-seven patients (43.1%) underwent an OAGB revision and sixty-two patients (56.9%) underwent an RYGB revision. Table 1 summarises the demographic and clinical characteristics of the two groups at baseline.

**Table 1:** Baseline characteristics

Variable	OAGB (n = 47)	RYGB (n = 62)	P-value
Age (years)	38.1 ± 9.2	40.7 ± 9.5	0.167
Sex (M/F)	M: 7 (14.9%), F: 40 (85.1%)	M: 10 (16.1%), F: 52 (83.9%)	1.000
Years from LSG to conversion	3.8 ± 1.3	4.1 ± 1.3	0.145
Height (cm)	162.2 ± 10.5	162.9 ± 6.9	0.699
Weight Before LSG (kg)	130.0 ± 27.8	128.7 ± 27.0	0.428
BMI Before LSG (kg/m^2^)	50.4 ± 8.5	48.2 ± 8.3	0.178
Lowest Weight after LSG (kg)	96.4 ± 20.3	90.1 ± 18.0	0.097
Lowest BMI after LSG (kg/m^2^)	36.6 ± 6.7	33.8 ± 6.1	0.028
Weight before conversion surgery (kg)	113.8 ± 20.1	107.2 ± 21.2	0.179
**BMI before conversion surgery (kg/m**^**2**^**)**	**43.3 ± 7.1**	**40.2 ± 6.5**	0.029*
ASA Score (I–IV)	2.1 ± 0.7	2.1 ± 0.6	0.735
Diabetes	6 (12.8%)	11 (17.7%)	0.658
Hypertension	7 (14.9%)	11 (17.7%)	0.730
Dyslipidaemia	4 (8.5%)	10 (16.1%)	0.206
Obstructive Sleep Apnoea (OSA)	0 (0.0%)	2 (3.2%)	0.087
Asthma	5 (10.6%)	7 (11.3%)	0.837
**GERD**	**3 (6.4%)**	**31 (50.0%)**	< 0.001*

The ages of OAGB and RYGB were similar (38.1 ± 9.2 vs. 40.7 ± 9.5, p = 0.167). There was no significant difference in sex distribution between the groups, with females representing predominance in both groups (85.1% in OAGB vs. 83.9% in RYGB, *p* = *1.000*). No significant differences were appreciated between the period of sleeve gastrectomy and conversion surgery (3.8 ± 1.3 vs. 4.1 ± 1.3 years, *p* = *0.145*). Similarly, the two groups had comparable height, post-LSG weight, and post-LSG BMI.

Regarding post-LSG outcomes, the lowest BMI attained (nadir BMI) in patients in the OAGB group was higher than that in patients in the RYGB group (36.6 ± 6.7 vs. 33.8 ± 6.1 kg/m^2^, *p* = *0.028*). The OAGB group had a significantly higher BMI before conversion surgery (43.3 ± 7.1 vs. 40.2 ± 6.5 kg/m2; *p* = *0.029*). All other parameters, such as ASA score and the presence of comorbidities such as diabetes, hypertension, dyslipidaemia, asthma, and obstructive sleep apnoea, were similar across groups. It is also important to note that the rate of pre-operative gastroesophageal reflux disease (GERD) was much higher in the RYGB conversion group compared to the OAGB conversion group (50.0% vs. 6.4%, *p* < *0.001*).

### Intraoperative and Postoperative Data

Table 2 presents the perioperative outcomes. The operative time was also significantly shorter for the OAGB group than it was for the RYGB group (median 77.5 min [IQR: 62.096.8] vs. 110.0 min [IQR: 90.0140.0], *p* < *0.001)*. There were no statistically significant differences in length of hospital stay between the groups (median 3.0 vs. 4.0 days, *p* = *0.280*). Intraoperative bleeding was also uncommon, occurring in one patient in each group (*p* = 1.000). There was one postoperative leak in the OAGB group; there were no leaks reported in the RYGB group (*p* = *0.431*).

**Table 2:** Intraoperative and Postoperative Data

Variable	OAGB (n = 47)	RYGB (n = 62)	p-value	N available*
Operative time (minutes)	77.5 [62.0–96.8]	110.0 [90.0–140.0]	** < 0.001**	OAGB: 46, RYGB: 60
Length of Hospital Stay (days)	3.0 [3.0–4.0]	4.0 [3.0–4.0]	0.280	OAGB: 46, RYGB: 62
Intraoperative Bleeding (yes/no)	1 (2.1)	1 (1.6)	1.000	OAGB: 47, RYGB: 62
Postoperative Leak (yes/no)	1 (2.1)	0 (0.0)	0.431	OAGB: 47, RYGB: 62

* “N available” refers to the number of patients with non-missing data for each variable

### Anthropometric Outcomes

Table 3 has the anthropometric results at 1-, 5-, and 7-year post-conversion operation. Both groups experienced significant weight loss in one year postoperatively. Absolute weight and BMI did not differ significantly between OAGB and RYGB. Nonetheless, the %TWL was much higher in the OAGB group (18.76 ± 9.12 vs. 15.04 ± 8.44, *p* = *0.032*) and the BMI decrease was also greater (8.24 ± 4.65 vs. 6.07 ± 3.44, *p* = *0.006*).

**Table 3:** Anthropometric Outcomes Comparison of OAGB versus RYGB after Sleeve Gastrectomy

Variable	OAGB mean ± SD	RYGB mean ± SD	p-value
BMI before conversion surgery (kg/m^2^)	43.31 ± 7.08	40.22 ± 6.46	**0.019**
Regained Weight (kg) before conversion	17.43 ± 11.49	22.90 ± 22.56	0.131
Weight (kg) at 1 year	92.23 ± 16.48	89.61 ± 17.99	0.443
BMI (kg/m^2^) at 1 year	35.50 ± 6.44	33.73 ± 5.90	0.218
%EWL at 1 year	47.79 ± 24.34	43.47 ± 27.49	0.466
%TWL at 1 year	18.76 ± 9.12	15.04 ± 8.44	**0.032**
BMI Reduction at 1 year	8.24 ± 4.65	6.07 ± 3.44	**0.006**
Weight (kg) at 5 years	96.07 ± 19.10	92.15 ± 19.27	0.300
BMI (kg/m^2^) at 5 years	36.65 ± 7.29	34.94 ± 6.07	0.129
%EWL at 5 years	36.78 ± 11.39	34.49 ± 14.47	0.785
%TWL at 5 years	15.08 ± 13.31	12.49 ± 10.67	0.270
BMI Reduction at 5 years	6.81 ± 5.07	5.13 ± 4.05	0.1103
Weight (kg) at 7 years	91.38 ± 18.11	92.17 ± 20.48	0.839
BMI (kg/m^2^) at 7 years	35.53 ± 7.35	34.55 ± 6.05	0.673
%EWL at 7 years	41.75 ± 11.78	37.43 ± 12.85	0.627
%TWL at 7 years	18.76 ± 13.07	13.23 ± 12.28	**0.028**
BMI Reduction at 7 years	8.36 ± 6.24	5.44 ± 4.96	**0.008**

At five years, the weight, BMI, and the %EWL and %TWL showed no statistically significant differences, and the BMI was reduced. At seven years, disparities were noted once more in support of OAGB. Although the absolute weight and the BMI were similar, OAGB patients exhibited a significantly higher %TWL (18.76 ± 13.07 vs. 13.23 ± 12.28, p = 0.028) and greater BMI reduction (8.36 ± 6.24 vs. 5.44 ± 4.96, p = 0.008). These results suggest that OAGB has better weight loss maintenance over time than RYGB.

### Comorbidity Outcomes

Table 4 summarizes the comorbidity outcomes after conversion surgery. In both groups, diabetes remission was limited. It did not differ significantly. In fact, only one patient in each group experienced complete diabetic remission (2%). The proportions of improvement were the same (p = 0.39). Hypertension remission was also rare, with isolated cases of complete remission or improvement in both groups (p = 0.29). The dyslipidemia results were similar, with no significant differences in remission or improvement observed (p = 0.60).

**Table 4:** Comorbidity Outcomes Comparison of OAGB versus RYGB after Sleeve Gastrectomy

Variable	OAGB	RYGB	p-value
Diabetes: Complete Remission	1 (2%)	1 (2%)	0.39
Improved	4 (9%)	3 (5%)	0.39
No Changes/Worse	1 (2%)	6 (10%)	0.39
Hypertension: Complete Remission		1 (2%)	0.29
Improved	1 (2%)	0 (0%)	0.29
No Changes/Worse	3 (6%)	8 (13%)	0.29
Dyslipidemia: Complete Remission	2 (4%)	4 (6%)	0.6
Improved	0 (0%)	2 (3%)	0.6
No Changes/Worse	2 (4%)	2 (3%)	0.6
OSA—Complete Remission	0 (0%)	2 (3%)	0.21
Improved	47 (100%)	60 (97%)	0.21
Asthma: Complete Remission	1 (2%)	0 (0%)	0.71
Improved	0 (0%)	1 (2%)	0.71
No Change/Worse	3 (6%)	2 (3%)	0.71
**GERD: Complete Remission**	**1 (2%)**	**13 (21%)**	**0.002**
**Improved**	**0 (0%)**	**6 (10%)**	**0.002**
**No Changes/Worse**	**7 (15%)**	**6 (10%)**	**0.002**

Regarding obstructive sleep apnea, two RYGB group participants achieved full remission; this was not seen in the OAGB group (p = 0.21). Improvement rates were high in both groups (100% for OAGB and 97% for RYGB). The results for asthma were low overall. In regard to GERD, there was a significant difference observed: the RYGB group resulted in a much higher remission rate and improvement compared with that of the OAGB group. Specifically, 21% of patients were in full remission, and 10% showed improvement. However, only 2% of the patients in the OAGB group experienced any improvement of GERD (p = 0.002). These findings suggest that RYGB may be more effective than OAGB in managing post-sleeve GERD.

### Complications and Nutritional Outcomes

Postoperative complications were generally uncommon and failed to show any significant differences when compared with each other. (Table 5) Bleeding, leaks, marginal ulcers, internal hernias, dumping syndrome, and malnutrition rates did not differ significantly.

**Table 5:** Comparison of Complications Outcomes Following OAGB versus RYGB

Variable	OAGB Mean ± SD	RYGB Mean ± SD	p-value
Bleeding (Yes = 1, No = 0)	0.021 ± 0.146	0.016 ± 0.127	0.8475
Leak (Yes = 1, No = 0)	0.021 ± 0.146	0.000 ± 0.000	0.3225
Marginal Ulcer (Yes = 1, No = 0)	0.064 ± 0.247	0.065 ± 0.248	0.9886
Internal Hernia (Yes = 1, No = 0)	0.000 ± 0.000	0.032 ± 0.178	0.1590
Bowel Obstruction (Yes = 1, No = 0)	0.000 ± 0.000	0.000 ± 0.000	NA
Dumping Syndrome (Yes = 1, No = 0)	0.085 ± 0.282	0.145 ± 0.355	0.3275
Stricture (Yes = 1, No = 0)	0.000 ± 0.000	0.000 ± 0.000	NA
Vitamin Deficiencies	0.170 ± 0.380	0.290 ± 0.458	0.1377
Severe Malnutrition (Yes = 1, No = 0)	0.000 ± 0.000	0.016 ± 0.127	0.3213
Iron Deficiency Anemia	0.574 ± 0.500	0.435 ± 0.500	0.1536

Nutritional complications were predominant. There was a slightly higher rate of vitamin deficiencies in the RYGB group (29% vs. 17% in OAGB), although this difference was not statistically significant (p = 0.138). Similarly, iron-deficiency anaemia was observed in both groups, with a tendency toward higher prevalence in OAGB (57.4% vs. 43.5%, p = 0.154). Severe malnutrition was also uncommon and reported in only one patient in the RYGB group.

**Fig. 1:**
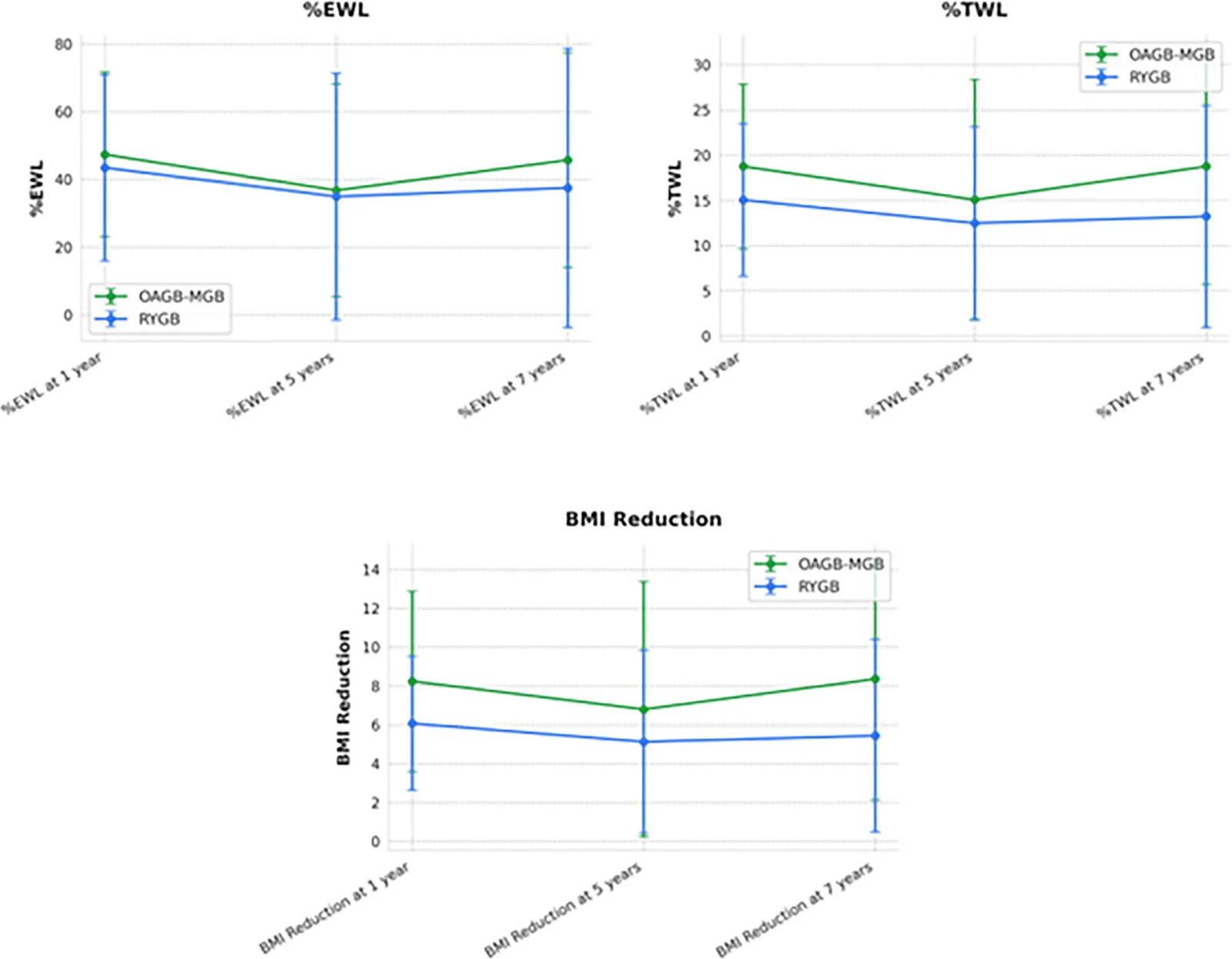
Anthropometric Outcomes of OAGB Compared to RYGB After SG

In summary, both conversional procedures had similar safety profiles; however, the OAGB group achieved better long-term weight loss, whereas the RYGB group experienced better GERD relief (Fig. 1).

During the 7-year follow-up, some late complications occurred. These included biliary reflux requiring conversion from OAGB to RYGB (n = 2), persistent GERD requiring laparoscopic vagotomy after RYGB (n = 1), and other late events such as incisional hernia (n = 2), marginal ulcer after OAGB (n = 2), chronic abdominal pain after RYGB (n = 1), and gallstone disease (n = 4; two in each group). These complications were successfully managed either surgically or medically. There were no cases of severe malnutrition or death recorded during follow-up.

## Discussion

This study compares the OAGB and the RYGB as conversion procedures in patients who have a previous SG. The results contribute to the limited research on long-term outcomes after five years. Our findings demonstrated significant differences in weight-loss sustainability, GERD resolution, and nutritional deficiencies. The OAGB conversion group demonstrated better long-term weight-loss outcomes. However, the RYGB conversion demonstrated itself to be more effective at managing post-sleeve GERD.

Baseline BMI difference was expected, as procedure selection was clinically driven rather than randomized. In general, patients with prominent GERD symptoms underwent RYGB; however, those who mainly complained of weight regain were offered OAGB. This resulted in a higher baseline BMI in the OAGB group.

### Long-Term Weight Loss Outcomes

Both procedures demonstrated impressive results as conversional options after “failed” sleeve gastrectomy. Nonetheless, OAGB patients experienced a significantly higher percentage of total weight loss (%TWL) and BMI reduction than RYGB patients at one and seven years, despite a higher BMI at the time of conversion.

These results align with other mid-term studies that have shown greater weight loss after OAGB compared to RYGB. This difference can be explained by the longer biliopancreatic limb and by the higher proportion of malabsorption in OAGB. According to the literature, OAGB not only is shown to result in shorter operative time but also exhibits stronger metabolic effects due to its intestinal bypass design [17, 18]. Our data further elaborates on these findings by demonstrating the long-term weight loss benefits of OAGB up to seven years. Although 7-year follow-up data are infrequently published, they provide relevant information on long-term weight maintenance and metabolic outcomes of conversional bariatric surgeries beyond 5 years. Furthermore, our findings are consistent with recent data demonstrating superior mid- to long-term weight loss after OAGB compared to RYGB in both primary and conversion settings. The improved weight durability observed in the OAGB cohort may be related to the longer biliopancreatic limb length, which is associated with a greater malabsorptive effect, as suggested in recent literature [19, 20].

### Resolution of Comorbidities

In this cohort, the influence of conversion procedures on obesity-related comorbidities was less pronounced compared to their effect on weight-loss outcomes. There were low rates of complete remission or improvement in diabetes, hypertension, dyslipidaemia, obstructive sleep apnea, or asthma in both groups; no significant differences were observed. This is partly due to the relatively low prevalence of these conditions in the study population, which reduces the statistical power to detect meaningful differences.

Conversely, there was a clear difference in GERD outcomes between the two procedures. RYGB resulted in significantly higher rates of GERD remission and improvement compared to OAGB. This aligns with the well-established practice that RYGB is the preferred procedure for patients with severe or refractory GERD. The mechanism involves diverting bile reflux and reducing oesophageal exposure to gastric acid by virtue of the Roux limb configuration. Conversely, OAGB has been reported to be associated with an increased risk of bile reflux, which may explain why GERD was not effectively resolved in this cohort [18, 21].

These findings emphasize the importance of selecting the procedure based on each individual case. For patients with SG and complications from GERD, RYGB seems to be a more effective conversion treatment, whereas OAGB may be a more suitable option for those without reflux disease who have experienced significant weight regain.

With respect to metabolic comorbidities, our results align with previously published data showing limited metabolic benefit of conversion surgery in the absence of severe baseline T2DM or hypertension. The superiority of RYGB for GERD resolution also parallels contemporary findings supporting RYGB as the preferred conversion option in patients with post-sleeve reflux [22, 23].

### Perioperative and Safety Outcomes

Regarding perioperative outcomes, OAGB conversions had shorter operative times than RYGB conversions. This is likely due to the OAGB configuration being somewhat technically simpler with its single-anastomosis design. This has been repeatedly observed in previous studies and may potentially lower the risk of surgery, especially in patients with multiple comorbidities or at high-volume surgical centers [24, 25].

Both groups experienced short hospital stays, although hospitalization was shorter in the OAGB group. However, the study was not powered to evaluate hospital stay or cost-related outcomes. There was minimal intraoperative bleeding, low postoperative leak rates, and no significant differences in major surgical complications.

### Nutritional Deficiencies and Long-Term Complications

Nutrition outcomes remain a significant concern after conversion bariatric surgery, particularly after malabsorptive procedures. We found a tendency for increased vitamin deficiencies in RYGB relative to OAGB, yet it failed to show statistical significance. On the other hand, iron deficiency anaemia was more common in the OAGB group. This makes sense as a result of the OAGB’s more malabsorptive tendencies [25].

Marginal ulceration, dumping syndrome, and internal hernia occurred at low rates and showed no significant differences between the groups. There was also a low incidence of severe malnutrition. These findings support the long-term safety of both conversion options discussed. However, OAGB is slightly more likely to lead to nutrient malabsorption, necessitating close long-term nutritional monitoring and supplementation [26].

### Clinical Implications

These results highlight the complementary strengths of OAGB and RYGB when used as sleeve conversion procedures. Conversional OAGB appears to be more effective for long-term weight loss and has shorter operative times; long-term effectiveness has been demonstrated to extend beyond seven years. On the other hand, RYGB conversions are advantageous in resolving GERD and should be prioritized for patients with reflux symptoms or post-SG complications. Clinically, the findings suggest that OAGB conversions can be suitable options for patients aiming for sustained weight loss, particularly those with a higher preoperative BMI or those who have experienced significant weight regain following SG [27, 28].

Clinically, the selection of conversion procedure must be customised, considering patient factors such as baseline BMI, the presence of GERD, comorbidity profile, and adherence to nutritional follow-up. Maximising the long-term outcomes through shared decision-making between patients and surgeons is necessary.

## Limitations

Because the study sample was determined by the number of eligible patients treated during the predefined timeframe, the study may be underpowered to detect small between-group differences. This is an inherent limitation of retrospective designs, and statistical power is therefore constrained by feasibility rather than formal sample size calculation. The retrospective design introduces the potential for selection bias, in which pre-existing GERD or BMI may have influenced the choice of procedure. As a result, baseline characteristics—particularly BMI and GERD status—were not matched between groups. The study may also be limited by its single-centre design, and the relatively low prevalence of comorbidities in the study population could reduce the ability to detect differences in disease resolution. Furthermore, the assessment of nutritional outcomes focused mainly on deficiency levels, without comprehensive, longitudinal monitoring of micronutrients, which could have overlooked subtle differences in metabolism. The patient's quality of Life (QoL) assessment was not included, which limits the evaluation of subjective well-being, functional status, and overall patient satisfaction.

## Conclusion

In summary, this seven-year comparative study demonstrates that both OAGB and RYGB conversion procedures after SG are effective, with durable weight loss at 7 years. However, OAGB conversion for weight recurrence regain after SG may yield better long-term weight-loss outcomes than RYGB conversion. Nevertheless, RYGB might better manage post-SG GERD. Neither procedure posed any danger and complications were minimal. Nutritional deficiencies were easily controlled. Conversion surgery, therefore, must be an individual decision. Weight loss and reflux control need to be balanced for each patient. However, given the limited sample size and retrospective nature of this study, these findings should be interpreted with caution. Further studies involving larger cohorts are needed to validate these results with the goal to better guide patient selection criteria for each conversion procedure.

## Data Availability

No datasets were generated or analysed during the current study.
